# CFTR Depletion Results in Changes in Fatty Acid Composition and Promotes Lipogenesis in Intestinal Caco 2/15 Cells

**DOI:** 10.1371/journal.pone.0010446

**Published:** 2010-05-05

**Authors:** Geneviève Mailhot, Rémi Rabasa-Lhoret, Alain Moreau, Yves Berthiaume, Emile Levy

**Affiliations:** 1 Research Centre, CHU Sainte-Justine, Université de Montréal, Montreal, Quebec, Canada; 2 Department of Nutrition, Université de Montréal, Montreal, Quebec, Canada; 3 Department of Pediatrics, Université de Montréal, Montreal, Quebec, Canada; 4 Cystic Fibrosis Clinic, Centre Hospitalier de l'Université de Montréal (CHUM)-Hôtel-Dieu, Montreal, Quebec, Canada; 5 Diabetes and Metabolic Diseases Research Group, Institut de Recherches Cliniques and Centre Hospitalier de l'Université de Montréal (CHUM)-Hôtel-Dieu, Université de Montréal, Montreal, Quebec, Canada; Emory Unviersity, United States of America

## Abstract

**Background:**

Abnormal fatty acid composition (FA) in plasma and tissue lipids frequently occurs in homozygous and even in heterozygous carriers of cystic fibrosis transmembrane conductance regulator (CFTR) mutations. The mechanism(s) underlying these abnormalities remained, however, poorly understood despite the potentially CFTR contributing role.

**Methodology/Principal Findings:**

The aim of the present study was to investigate the impact of CFTR depletion on FA uptake, composition and metabolism using the intestinal Caco-2/15 cell line. shRNA-mediated *cftr* gene silencing induced qualitative and quantitative modifications in FA composition in differentiated enterocytes as determined by gas-liquid chromatography. With the *cftr* gene disruption, there was a 1,5 fold increase in the total FA amount, largely attributable to monounsaturated and saturated FA compared to controls. The activity of delta-7 desaturase, estimated by the 16:1(n-7)/16:0, was significantly higher in knockdown cells and consistent with the striking elevation of the n-7 FA family. When incubated with [^14^C]-oleic acid, CFTR-depleted cells were capable of quick incorporation and export to the medium concomitantly with the high protein expression of L-FABP known to promote intracellular FA trafficking. Accordingly, lipoprotein vehicles (CM, VLDL, LDL and HDL), isolated from CFTR knockdown cells, exhibited higher levels of radiolabeled FA. Moreover, in the presence of [^14^C]-acetate, knockdown cells exhibited enhanced secretion of newly synthesized phospholipids, triglycerides, cholesteryl esters and free FA, thereby suggesting a stimulation of the lipogenic pathway. Conformably, gene expression of SREBP-1c, a key lipogenic transcription factor, was increased while protein expression of the phosphorylated and inactive form of acetylCoA carboxylase was reduced, confirming lipogenesis induction. Finally, CFTR-depleted cells exhibited lower gene expression of transcription factors (PPARα, LXRα, LXRβ and RXRα).

**Conclusions/Significance:**

Collectively, our results indicate that CFTR depletion may disrupt FA homeostasis in intestinal cells through alterations in FA uptake and transport combined with stimulation of lipogenesis that occurs by an LXR/RXR-independent mechanism. These findings exclude a contributing role of CFTR in CF-associated fat malabsorption.

## Introduction

The basic defect in cystic fibrosis (CF) is caused by mutations in the epithelial chloride channel, known as the cystic fibrosis transmembrane conductance regulator (CFTR). Nearly 1500 CFTR mutations have been identified insofar, which contribute to create a wide spectrum of disease severity. The most common mutation remained the deletion of a phenylalanine residue at position 508 (ΔF508), which causes inappropriate folding of CFTR, followed by its proteolytic degradation in the endoplasmic reticulum. This mutation is present in nearly 70% CF patients while 4% of the general population is estimated to be heterozygous carriers of ΔF508. Manifestations of the disease include pancreatic insufficiency, intestinal fat malabsorption and chronic lung infections that ultimately lead to pulmonary failure and death [1].

It is widely recognized that disturbances in plasma and tissue fatty acid (FA) profile constitute a persistent feature of CF. Multiple studies performed on cellular [2], [3] and animal models [4] of CF as well as on CF-affected individuals and obligate heterozygous [5] have shown alterations in FA composition, particularly in the n-6 and n-3 polyunsaturated FA (PUFA). Decreased levels of linoleic acid (LA; 18:2n-6), docosahexaenoic acid (DHA; 22:6n-3) and normal to increased levels of arachidonic acid (AA; 20:4n-6) have frequently been reported in CF plasma, cells and tissues [5]–[7]. These abnormalities occur irrespective of the amount of energy and fat ingested or the pancreatic status, which argues against a nutritional origin and appears more suggestive of abnormalities in FA metabolism [8], [9]. Further studies are, however, needed in order to explore the specific mechanism(s).

Intestinal mucosa represents a tissue particularly sensitive to changes in FA environment [8]. Enterocyte cell membranes are particularly rich in LA and AA, and alterations in FA composition affect membrane fluidity and function of membrane proteins, modify intracellular phases of fat absorption and influence eicosanoid production and inflammatory processes [10]–[12]. Importantly, CF patients are instructed to consume a high-energy, high-fat diet to minimize the deleterious effects of fat malabsorption on health [13]. Therefore, intestinal cells are consistently exposed to high luminal concentrations of FA. With the exception of one study reporting increased AA and reduced DHA levels in the ileum of CFTR knockout mice [4], very limited data are available on intestinal FA composition and metabolism in relation to CF or CFTR function despite evidence of essential FA deficiency, intestinal inflammation and abnormalities in enterocyte lipid trafficking in CF [14]–[18].

Classically, lipid malabsorption and steatorrhea have been recognized as secondary manifestations of pancreatic insufficiency and intra-duodenal acidification [19]. However, a recent work characterizing post-lipolytic events in duodenal explants of CF patients has found defects in the intracellular phase of lipid transport [15]. These findings have raised the possibility that other factors independently of pancreatic function and luminal environment might contribute to irregular lipid transport in CF small intestine. Evidence in support of this hypothesis comes from the clinical observation that pancreatic enzyme supplementation coupled to anti-acid therapy could not correct malabsorption and steatorrhea in CF patients [20], [21]. Other studies have reported a possible link between CFTR function and essential FA utilization [3], [22] but have focused on various models of epithelial cells other than intestinal cells. Recent work in our lab has addressed the consequences of reduced CFTR expression on the handling, transport and secretion of lipids by the intestinal Caco-2/15 cell line and has shown that CFTR disruption exerted a stimulated action on intestinal lipid synthesis and transport [23]. This inverse relationship between CFTR expression and intestinal lipid metabolism indicated that the primary gene defect is not responsible for the persistent fat malabsorption in CF patients although variation in CFTR protein expression appears to regulate lipid homeostasis. In line with our previous work and considering the possible interaction between CFTR function and FA homeostasis, we carried out studies to examine the impact of CFTR depletion on FA uptake, composition and metabolism in the intestinal Caco-2/15 cell line.

## Methods

### Cell culture

The colon carcinoma cell line, Caco-2/15 (ATCC, Rockville, MD), was cultured at subconfluent stages in MEM supplemented with 5% fetal bovine serum, 1% non-essential amino acids and 1% Penicillin-Streptomycin (all reagents from Gibco, Grand Island, NY) as described before [24]. For several experiments, 1×10^6^ cells were seeded on 24.5-mm polycarbonate Transwell filter inserts (Costar, Cambridge, MA) with 0.4-µm pores to allow separate access to the upper and lower compartments of the monolayer. Cells were cultured for 12 to 15 days, which represented an appropriate period to study lipid metabolism in state of full differentiation [25], [26]. Cell differentiation was assessed by determination of villin protein expression and the integrity of Caco-2/15 monolayers was confirmed by transepithelial resistance measurement as described previously [27].

In a subset of experiments, differentiated Caco-2/15 cells were treated for 3 days with 20 µM of the highly selective CFTR inhibitor, CFTR-inh172 (Sigma, St-Louis, MO). In order to maintain continuous CFTR inhibition, culture medium, including CFTR-inh172 was replenished every 24 h. Cells treated with 0.1% DMSO, the vehicle used to dissolve the inhibitor, served as controls.

### Virus production and cell infection

shRNAs targeting CFTR and individually cloned into plko.1-puromycin vector (Open Biosystems, Huntsville, AL) were used to generate lentiviral particles in HEK293FT packaging cells. Cells were transfected in suspension using the standard calcium-phosphate method with 5 ug of each of the following DNA plasmids: pLP1, pLP2, pLP/VSVG and either plko.1 empty vector, plko.1-containing CFTR shRNA insert or pLentiV5–green fluorescent protein. The medium was changed 24 h post-transfection for antibiotic-free DMEM supplemented with 10% fetal bovine serum, 1% sodium pyruvate (Sigma, St-Louis, MO), 1% non-essential amino acids and 1% GlutaMAX™ (Gibco, Grand Island, NY). The viral supernatants were collected 3-days post-transfection, aliquoted and stored at −80°C. Virus titers were assessed with the Lenti-X qRT-PCR titration kit (Qiagen,Valencia, CA).

After trypsinization, 1×10^6^ Caco-2/15 cells were infected in suspension in the presence of 4 ug/mL of Polybrene (hexadimethrine bromide, Sigma, St-Louis, MO). Fresh culture medium was added 1 h post-infection. After a 3-day-incubation with lentivirus, cells were trypsinized, transferred to 10-cm^2^ dishes and allowed to grow in presence of the selection antibiotics, puromycin (Sigma, St-Louis, MO). When cells reached 80% confluence, they were plated at a density of 1×10^6^cells on 24.5-mm polycarbonate Transwell filter inserts and allowed to differentiate in the presence of puromycin. Infection efficiency was assessed by the monitoring of green fluorescent protein expression in cells infected with lentivirus carrying the green fluorescent protein. Cells mock-infected with the empty vector plko.1 served as controls since preliminary experiments have shown comparable gene and protein CFTR expression between mock and non-treated cells.

### Fatty acid analysis

Following differentiation, Caco-2/15 cells were serum-starved for 24 h before being homogenized in PBS supplemented with 1 mM of anti-proteases and 0.01% butylated hydroxytoluene (BHT; Sigma, St-Louis, MO). Samples were subjected to transesterification and injected into a gas chromatograph using a 90 m×0.32 mm WCOT-fused silica capillary column VF-23ms coated with 0.25 µm film thickness (Varian, Canada) according to the method described previously [28].

### RNA isolation and RT-PCR

Total RNA was extracted from differentiated Caco-2/15 cells using Trizol (Invitrogen, Carlsbad, CA) and complementary DNA generated using the Moloney Murine Leukemia Virus reverse transcriptase (Invitrogen, Carlsbad, CA) according to manufacturer's instructions. PCR reactions for CFTR, transcription factors [(liver X receptors (LXRs), retinoid X receptors (RXRs), peroxisome proliferators-activated receptors (PPARs) and sterol regulatory element binding protein-1c (SREBP-1c)] and GAPDH (as a house-keeping gene) were set up using intron-spanning primers and performed on a GeneAmp PCR System 9700 (Applied Biosystems, Foster City, CA) as described previously [29]. Approximately 26 to 35 cycles of amplification were used for each target at 95°C for 30 seconds, 53–62°C for 1 min and 72°C for 1 min. The number of cycles corresponds to the linear portion of the exponential phase for each gene expression and was assessed in preliminary studies.

### Immunoblotting

Mock-infected and CFTR knockdown cells were homogenized after 14 days of differentiation and prepared for Western blot as described before [30]. Briefly, 20 µg of total proteins were fractionated on a SDS-PAGE gel and blotted onto nitrocellulose membrane. After a blocking step consisting of a short incubation (1 h) with 5% of nonfat milk, membranes were probed overnight with 1∶5000 anti-Fatty acid translocase/cluster determinant 36 (FAT/CD36; Santa Cruz, Santa Cruz, CA), 1∶1000 anti-L-fatty acid binding protein (L-FABP; prepared in our lab), 1∶1000 anti-CFTR, acetyl-CoA carboxylase (ACC), AMP-activated protein kinase (AMPK), phosphorylated ACC or phosphorylated AMPK (Cell Signaling, Danvers, MA) or 1∶40 000 anti-β-actin (Sigma, St-Louis, MO) followed by a 1-h-incubation with species-specific horseradish peroxidase-conjugated secondary antibodies (at a 1∶10000 dilution) and revealed using the chemiluminescent reagent Luminol (Roche Diagnostics, Mannheim, Germany). The films were quantified by computer-assisted scanning densitometry using UN-SCAN-IT software (Silk Scientific, Orem, UT).

### Isolation and analysis of intracellular lipid fractions and lipoproteins

Caco-2/15 cells were differentiated for 10 days on polycarbonate Transwell filter inserts as described above. Lipid synthesis was determined as described previously [24]. On day 10, fetal bovine serum was omitted from the cell culture medium and cells were preincubated with 1 µmol/mL of cold oleic acid. After a 24-h preincubation, 0.45 µCi/well of [^14^C]-oleic acid (specific activity of 55 mCi/mmol; GE Healthcare, Piscataway, NJ) was added in the upper compartment of the Transwell for another 24-h period. Basolateral media were subsequently collected and cells were rinsed twice in ice-cold PBS and lysed in a buffer consisting of 10 mM Tris, 150 mM NaCl, 0.025% sodium azide, 5 mM EDTA, 0.1% SDS, 1% Triton X100 and 0.5% sodium desoxycholate together with 1 mM of each antiprotease: pepstatin A, PMSF and aprotinin. Cell protein was quantified by the Bradford method (BioRad, Hercules, CA). Cell homogenates and basolateral media were lipid-extracted with chloroform/methanol (2∶1, vol:vol) and lipids were separated by thin-layer chromatography (TLC) using the solvent mixture: hexane/ethyl ether/acetic acid (80∶20∶3, (vol:vol:vol)). The area corresponding to the free fatty acids (FFA) was excised from the TLC silica plates, transferred to scintillation vials with 10 mL of scintillation fluid (Beckman-Coulter, Fullerton, CA) and radioactivity measured. Quenching was corrected using computerized curves generated with external standards.

Lipoproteins were isolated from basolateral media by serial ultracentrifugation using a TL-100 ultracentrifuge (Beckman-Coulter, Fullerton, CA) according to the method described previously [31]. Briefly, chylomicrons (CM) were first isolated after an ultracentrifugation at 25 000 g for 40 min. Very-low-density lipoprotein (VLDL; 1.006 g/mL) and low-density lipoprotein (LDL; 1,063 g/mL) were subsequently separated by centrifugation at 100 000 g for 2.5 h. High-density lipoprotein (HDL) fraction was obtained after adjusting the LDL infranatant to a density of 1.21 g/mL and centrifuging for 6 h at 100 000 g. Each lipoprotein fraction was dialyzed against 0.15 M NaCl and 0.001 M EDTA, pH 7.0 at 4°C for 24 h. Lipids from each lipoprotein were extracted with chloroform/methanol and the FFA fraction analyzed as described in details above.

### Lipid carrier

Blood (20 mL) was collected by venipuncture 2–3 h after the ingestion of a high fat meal (50 g/1,73 m^2^) from two human healthy volunteers. This procedure was approved by the Institutional Ethic Committee. After a 1000×g centrifugation to pellet red blood cells, postprandial plasma was supplemented with 1 mM of aprotinin and 0.1% of sodium azide and was mixed with basolateral media to serve as a carrier for the isolation of labeled CM as described previously [24].

### De novo lipogenesis

Caco-2/15 cells were serum-starved after 12 days of differentiation on Transwell filter inserts. After 24-h-incubation in serum-free medium, cells were cultured in the presence of 5 µCi of sodium-[^14^C]-acetate (specific activity of 50–62 mCi/mmol; GE Healthcare, Piscataway, NJ) for the same length of time. Cells and media were collected, supplemented with a mixture of anti-proteases and lipids were extracted overnight in chloroform-methanol (2∶1, vol:vol). Lipids recovered were separated on TLC plates, and bands corresponding to phospholipids (PL), FFA, triglycerides (TG) and cholesteryl ester (CE) were scraped off the plates, mixed with scintillation fluid and counted for the amount of radioactivity incorporated as described previously [32].

### Statistical analysis

Data are presented as means ± SEM. To account for the inter-variability between experiments, some data are given as percentage of mock-infected cells. Student'*t* test was used for statistical analysis of differences between means and cut-off point for significance was set at *P*<0.05.

## Results

### Influence of CFTR knockdown on FA concentration and composition

**Figure 1:**
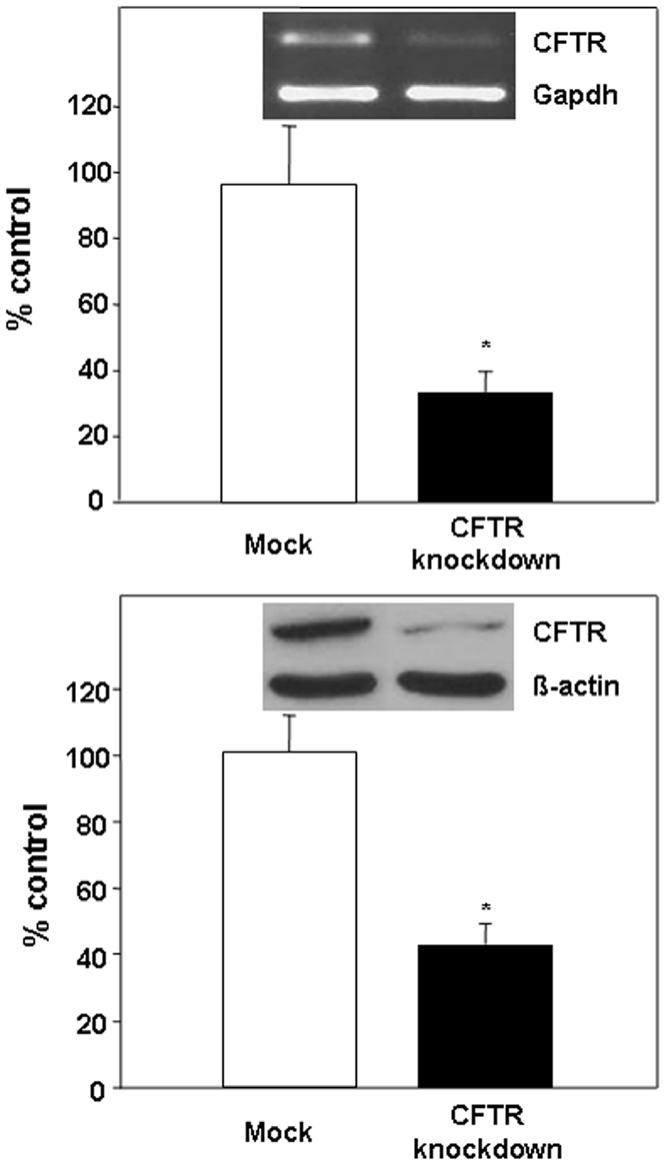
CFTR gene and protein knockdown following lentivirus infection of Caco 2/15 cells. After lentivirus infection, Caco 2/15 cells were differentiated for a period of 14 days before being assessed for CFTR gene and protein expression by RT-PCR and Western Blot, respectively. Cells that were not infected with lentivirus served as controls and represent 100%. Cells infected with the empty vector (indicated as Mock Cells on the graph) exhibited comparable CFTR expression relatively to noninfected cells. Results represent mean± SEM of n = 5 independent cell preparations and are illustrated as % of the noninfected cells after the data have been calculated as densitometric ratio of CFTR to GAPDH or β-actin. *p<0,01.

**Figure 2:**
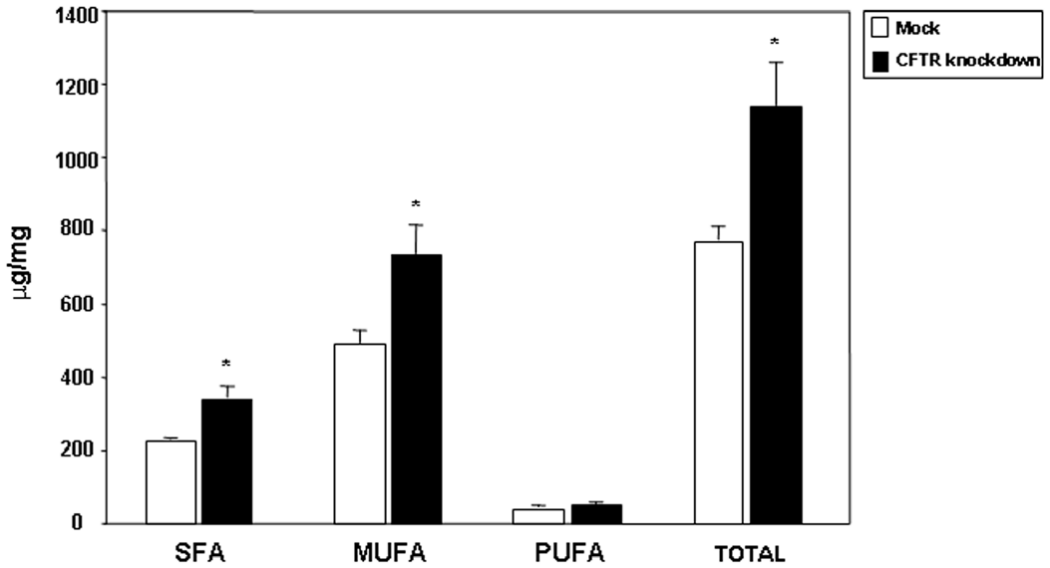
Influence of CFTR knockdown on total fatty acids (FA) content and FA classes in Caco-2/15 cells. At 15 days of differentiation, cells were collected, subjected to direct transesterification and injected into a gas chromatograph. Results represent the means ± SEM of 4 independent experiments and are illustrated as µg/mg of cellular protein. *p<0.05 vs mock cells. SFA: saturated fatty acids; MUFA: monounsaturated fatty acids; PUFA; polyunsaturated fatty acids.

We used the shRNAi experimental approach to generate a model of Caco-2/15 cells deficient in CFTR. Infection of Caco-2/15 cells with lentivirus carrying CFTR-shRNAi caused a reduction in CFTR gene (61%) and protein (57%) expression when compared to non-infected cells (Figure 1). Mock-infected cells expressed a level of CFTR gene and protein, which was similar to non-infected cells and were, therefore, used as controls in subsequent experiments. Cell viability, integrity and differentiation state were not altered by genetic manipulation (data not shown). FA composition of cell homogenates were assessed by gas chromatography and comparisons were set up between control mock and knockdown cells. Total amount of FA increased by 1.5-fold upon CFTR gene disruption (Figure 2). This raised level was imputed to increased levels of specific FA species, including monounsaturated (50%), and saturated (49%) FA relatively to mock-infected cells. In particular, the total n-7 FA levels (236.5±7.0 µM/mg protein vs 416.8±60.4 µM/mg protein) experienced the highest increase (76% over mock values) whereas total n-9 FA levels (252.7±25.2 µM/mg protein vs 319.4±19.4 µM/mg protein) were only moderately raised (26% over control values). The proportions of individual FA are summarized in Table 1. CFTR knockdown resulted in a marked increase in the amounts of myristic (14:0) and palmitic (16:0) FA, while stearic acid (18:0) and longer saturated FAs displayed reduced values (P<0.05). As a consequence of CFTR genetic manipulation, n-7 FAs appeared elevated whereas n-3 and n-6 FAs remained unchanged. Proportions of n-9 FA were reduced to various degrees in knockdown cells with oleic acid (18:1n-9) exhibiting the highest, albeit non-significant, decrease compared to mock cell values.

**Table 1:** Fatty acid composition in Caco-2/15 cells.

Fatty acids	Mock (%)	CFTR Knockdown (%)	*p*-Value
**14:0**	1.49±0.25	2.68±0.03	<0.05
**15:0**	0.72±0.01	1.63±0.16	<0.05
**16:0**	14.92±1.01	18.17±0.19	<0.05
**17:0**	0.70±0.00	0.77±0.10	
**18:0**	8.41±0.37	4.64±0.04	<0.05
**20:0**	0.53±0.03	0.27±0.01	<0.05
**22:0**	0.56±0.04	0.27±0.00	<0.05
**24:0**	1.36±0.06	0.62±0.03	<0.05
**18-3 (n-3)**	0.02±0.00	0.02±0.0	
**20:3 (n-3)**	0.01±0.00	0.01±0.00	
**20:5 (n-3)**	0.42±0.05	0.21±0.05	<0.05
**22:5 (n-3)**	0.33±0.01	0.40±0.03	
**22:6 (n-3)**	0.81±0.03	0.78±0.13	
**18:2 (n-6)**	0.63±0.06	0.46±0.06	
**20:2 (n-6)**	0.06±0.01	0.04±0.00	
**20:3 (n-6)**	0.36±0.04	0.21±0.04	
**20:4 (n-6)**	2.06±0.21	1.46±0.19	
**22:4 (n-6)**	0.15±0.01	0.56±0.40	
**16:1 (n-7)**	8.40±0.06	13.20±0.07	<0.05
**18:1 (n-7)**	22.29±0.66	23.21±1.50	
**18:1 (n-9)**	26.75±1.58	23.74±1.04	
**20:1 (n-9)**	1.68±0.03	1.33±0.01	<0.05
**22:1 (n-9)**	2.89±0.12	1.98±0.14	<0.05
**24:1 (n-9)**	1.27±0.05	0.97±0.00	<0.05
**Total n-3**	1.60±0.09	1.42±0.21	
**Total n-6**	3.37±0.32	2.78±0.09	
**Total n-7**	30.76±0.59	36.43±1.57	<0.05
**Total n-9**	32.75±1.68	28.16±1.18	

After 15 days of differentiation, cells were serum-starved and collected for fatty acid analysis. Cells were then subjected to transesterifiation and injected into a gas chromatograph. Results are expressed as % of total fatty acid content. Data represent the means±SEM of four independent wells.

The ratio of 16∶1 (n-7)/18∶2 (n-6), an established index of essential FA status [33], was increased by 2-fold in CFTR-depleted cells (Table 2). Similar trend was also observed for the 20∶3 (n-9)/20∶4 (n-6) and the DHA/AA ratio. Finally, the activity of delta-7 and delta-9 desaturases, estimated by the 16∶1(n-7)/16∶0 and the 18∶1(n-9)/18∶0 ratios, was significantly higher in CFTR knockdown cells than mock-infected cells whereas the delta-6 desaturase index, 20∶3 (n-6)/18∶2 (n-6), was reduced.

**Table 2:** Ratios and desaturation indexes in Caco-2/15 cells.

Fatty acid	Mock	CFTR Knockdown	*p*-Value
** 16:l (n-7)/18:2(n-6)**	13.48±1.26	29.04±3.78	<0.05
** 20:3(n-9)/20:4(n-6)**	0.073±0.004	0.093±0.005	<0.05
** EFA/non-EFA**	0.007±0.001	0.005±0.001	
** DHA/AA**	0.398±0.026	0.534±0.016	<0.05

Desaturation indexes			
**Δ6 20:3(n-6)/18:2 n-6**	0.564±0.005	0.442±0.026	<0.05
**Δ9 18:1 n-9/18:0**	3.195±0.329	5.111±0.177	<0.05
**Δ7 16:1 n-7/16:0**	0.566±0.042	0.726±0.004	<0.05

Student's *t* test (two-tailed) was used to compare differences between means (X±SEM).

EFA: essential fatty acids, AA: arachidonic acid, DHA: docosahexaenoic acid.

**Figure 3:**
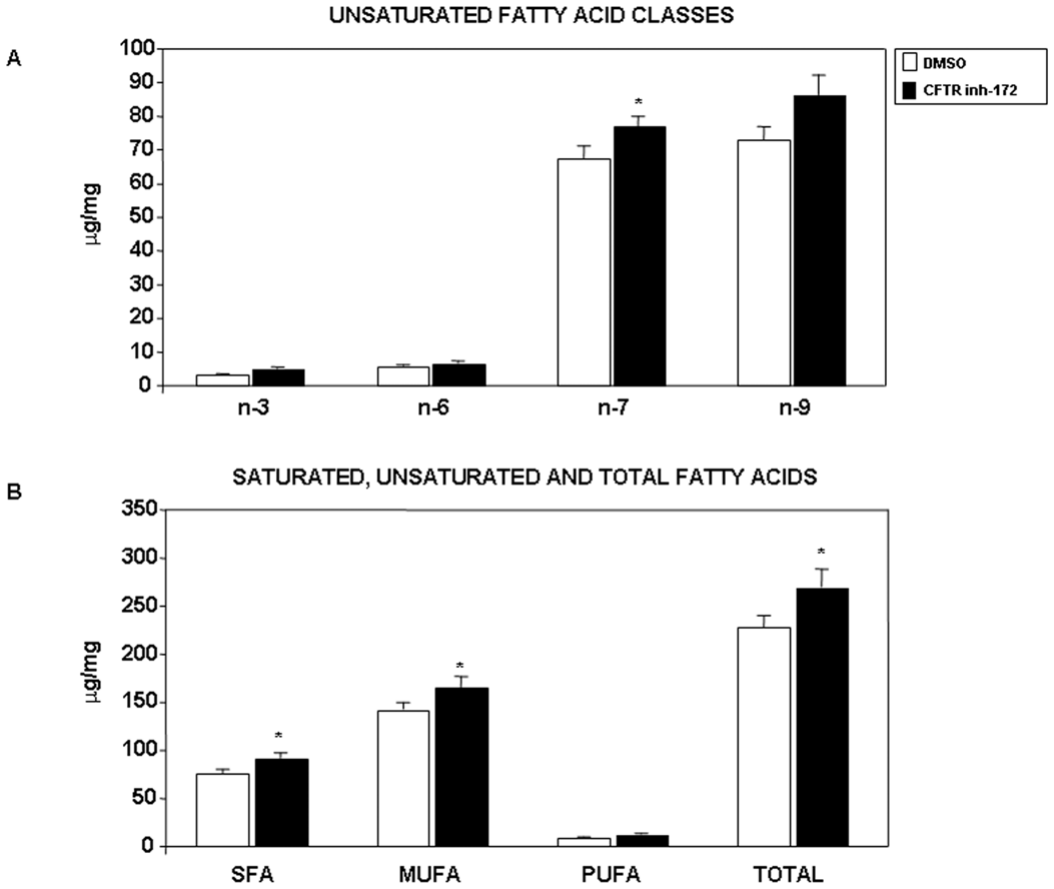
Influence of pharmacological inhibition of CFTR on fatty acid concentrations in Caco-2/15 cells. Treatment with 20 µM CFTR inh-172 at 12 days of differentiation was initiated and maintained for 3 days. Cells treated with DMSO (vehicle) served as controls. Cells were collected and subjected to direct trans-esterification and injected into a gas chromatograph. Result represent the means ± SEM of n = 3 to 4 separate wells originating from 3 independent experiments and are illustrated as µg/mg of cellular protein. *p<0.05 vs DMSO-treated cells. SFA: saturated fatty acids; MUFA: monounsaturated fatty acids; PUFA; polyunsaturated fatty acids.

To further confirm the influence of CFTR on FA concentration and composition, we examined the effect of the CFTR inhibitor, CFTR-inh172, that selectively blocks CFTR chloride conductance. In preliminary experiments, Trypan blue exclusion test and MTT assay were performed to assess cytotoxicity of this compound. Over 98% viability was recorded in cells treated with this chemical agent, and MTT values were not different between DMSO and CFTR-inh 172 treated cultures (data not shown). Pharmacological inhibition of CFTR led to modest but statically significant increase in the concentrations of saturated FA and monounsaturated FA, which resulted in a higher total FA content (Figure 3). Similarly to CFTR knockdown cells, CFTR-inh172-treated cells displayed a higher n-7 FA content and no significant alterations in the concentrations of n-3 and n-6 FAs. Besides, total n-9 FA exhibited a tendency toward increased values.

### Influence of CFTR knockdown on FA uptake and output

**Figure 4:**
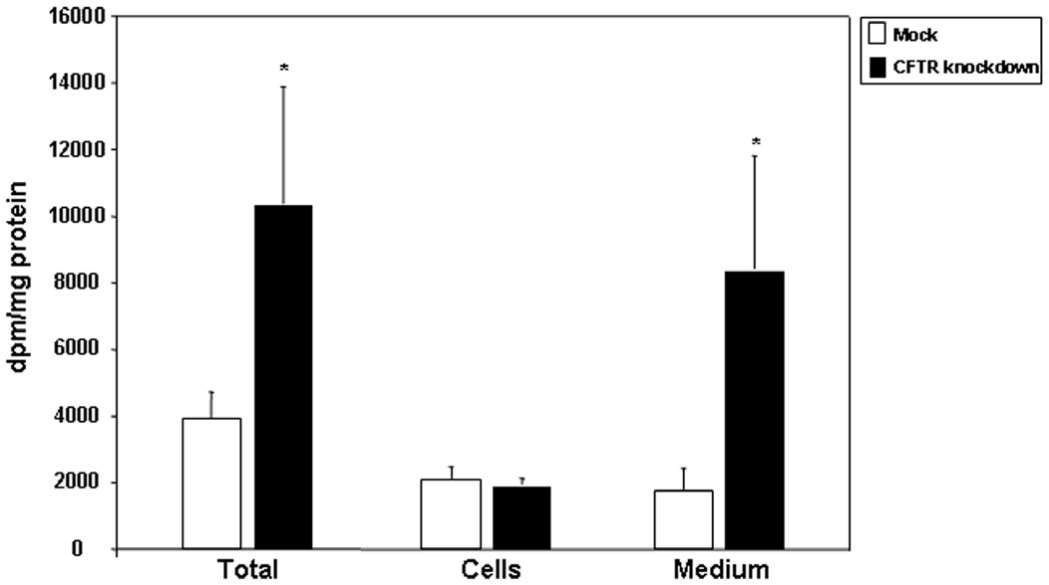
Total, cellular and medium free fatty acid (FFA) content of Caco-2/15 cells. Mock-infected and knockdown cells were differentiated for 12 days and incubated with [^14^C]-oleic acid for 24 h. Lipids of cell homogenates and medium were extracted with chloroform-methanol, isolated by TLC and the radioactivity incorporated into FFA fraction determined. Results were analyzed as dpm/mg of total protein but were reported as a proportion of mock-infected values representing 100%. Data represented means ± SEM of n = 3 independent experiments. *p<0.05 vs mock cells.

**Figure 5:**
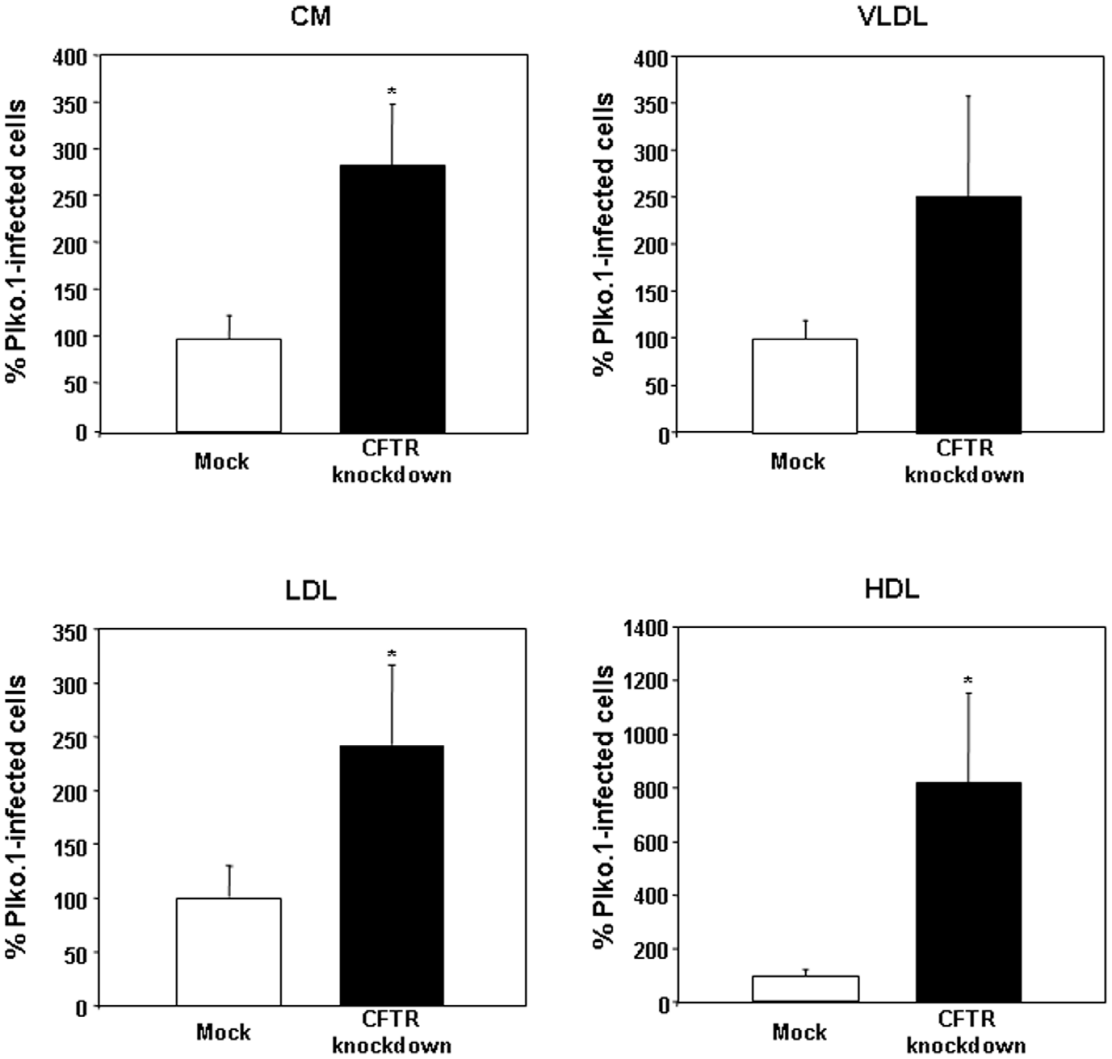
Free fatty acid (FFA) content in lipoproteins secreted by Caco-2/15 cells. Cells infected with empty vector plko.1 or infected with lentivirus carrying CFTR-shRNAi were differentiated for 12 days and incubated with [^14^C]-oleic acid for 24 h. Thereafter, lipoproteins were isolated by ultracentrifugation according to their specific densities. Lipoprotein lipids were further extracted using a mixture of chloroform/methanol, spreaded onto TLC plates and the band corresponding to the FFA fraction was scraped off the plates. Radioactivity incorporated was further determined. Data were analyzed as dpm/mg of total protein but were reported as percent difference relative to plko.1-infected values representing 100%. Data represented means ± SEM of n = 3 independent experiments. *p<0.05 vs mock cells. CM: chylomicron; HDL: high density lipoprotein; LDL: low density lipoprotein; VLDL: very-low density lipoprotein.

To verify whether changes in FA concentration might be accounted for by alterations in FA uptake and output by the intestinal cell, we incubated cells with a common dietary FA, [^14^C]-oleic acid, and determined the amount of radioactivity present in various cellular and secreted lipid fractions. CFTR knockdown cells displayed a 4-fold increase in the amount of FFA exported to the basolateral medium whereas no changes were recorded in the cellular amount of FFA (Figure 4). Subsequently, we measured the amount of FA transported by the lipoprotein classes: CM, VLDL, LDL and HDL isolated from CFTR knockdown cells. They displayed higher levels of FFA compared to control mock-infected cells: 186%, 153%, 143%, and 719%, respectively (Figure 5).

### Protein expression of transporters involved in FA uptake by intestinal cells

**Figure 6:**
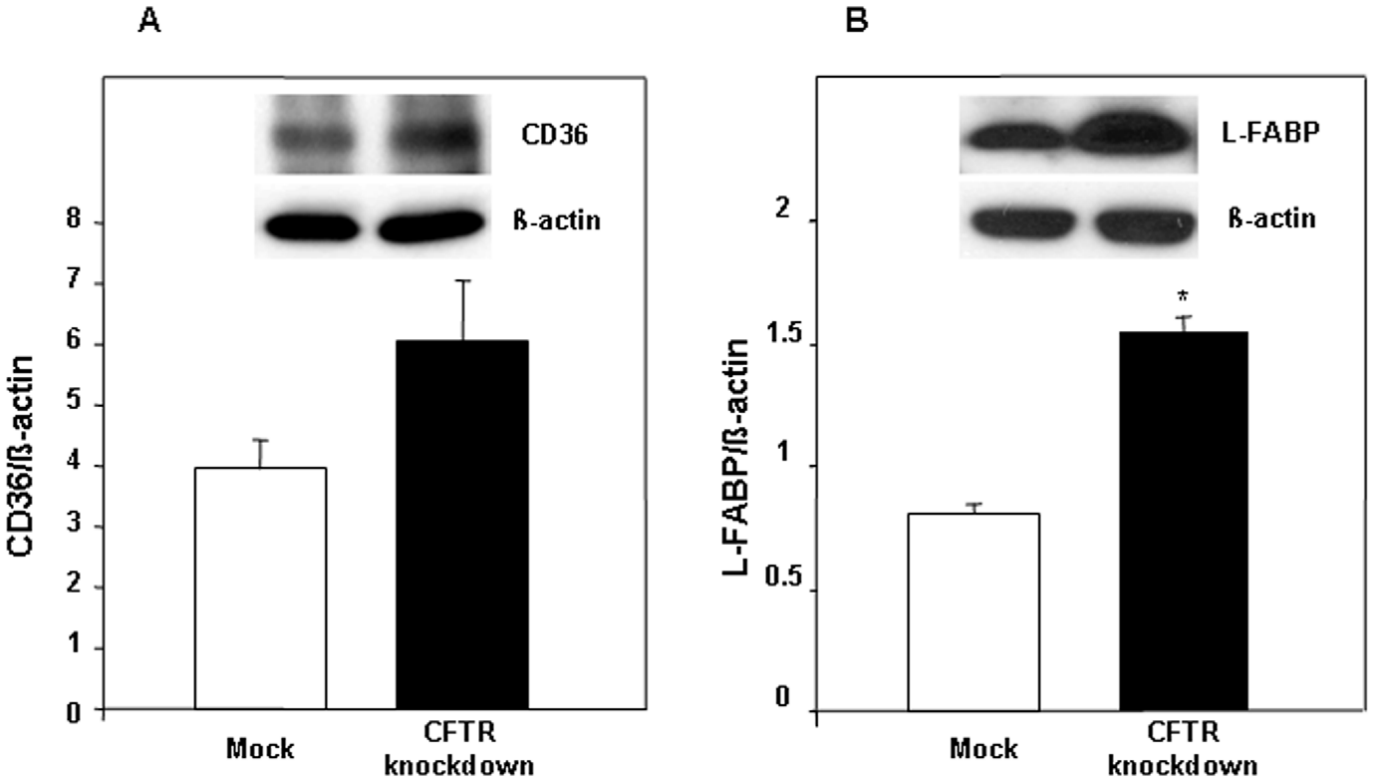
Effect of CFTR knockdown on protein expression of CD36 (A) and L-FABP (B). After 15 days of differentiation, Caco-2/15 cells were lysed and protein expression determined by Western Blot. β-actin served as loading control and was used for normalization. Data represented means ± SEM of three to four independent experiments *p<0.05 vs mock cells.

To elucidate the mechanisms underlying the significant raise of FA upon reduction in CFTR expression, we assessed the expression of two proteins involved in FA intestinal uptake and transport. First, the protein expression of FAT/CD36, a transporter involved in FA uptake, displayed a 1,5-fold increase in CFTR-depleted cells (Figure 6A) although this difference did not reach the significance threshold (p<0.09). Conversely, the protein expression of L-FABP that participates mainly in intracellular FA trafficking was increased by almost 2-fold in CFTR knockdown cells (Figure 6B).

### De novo lipogenesis

**Figure 7:**
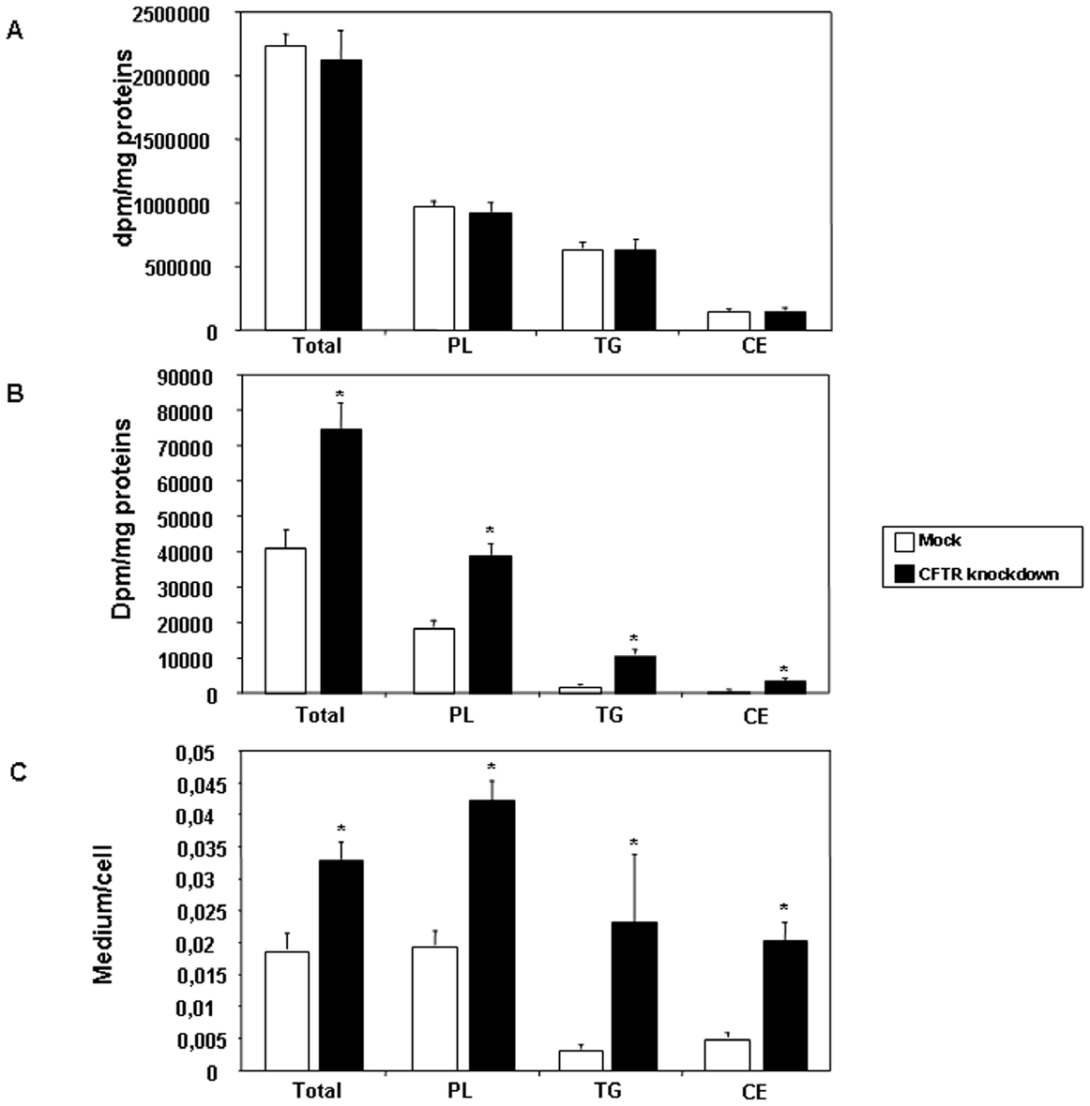
Influence of CFTR knockdown on *de novo* lipogenesis in Caco-2/15 cells. After 12 days of differentiation, cells were incubated with [^14^C]-acetate for 24 h. Cells (A) and media (B) were further collected and lipid-extracted in chloroform/methanol. The extracts were separated on silica plates and bands corresponding to TG, PL and CE were excised. Radioactivity incorporated into each fraction was counted. The discrepancy between synthesis of newly formed lipids and their secretion became evident when expressed as a medium/cell ratio (C). Data represented means ± SEM of n = 12 wells from two independent experiments. *p<0.05 vs mock cells. TG: triglycerides; PL: phospholipids; CE: cholesteryl ester.

**Figure 8:**
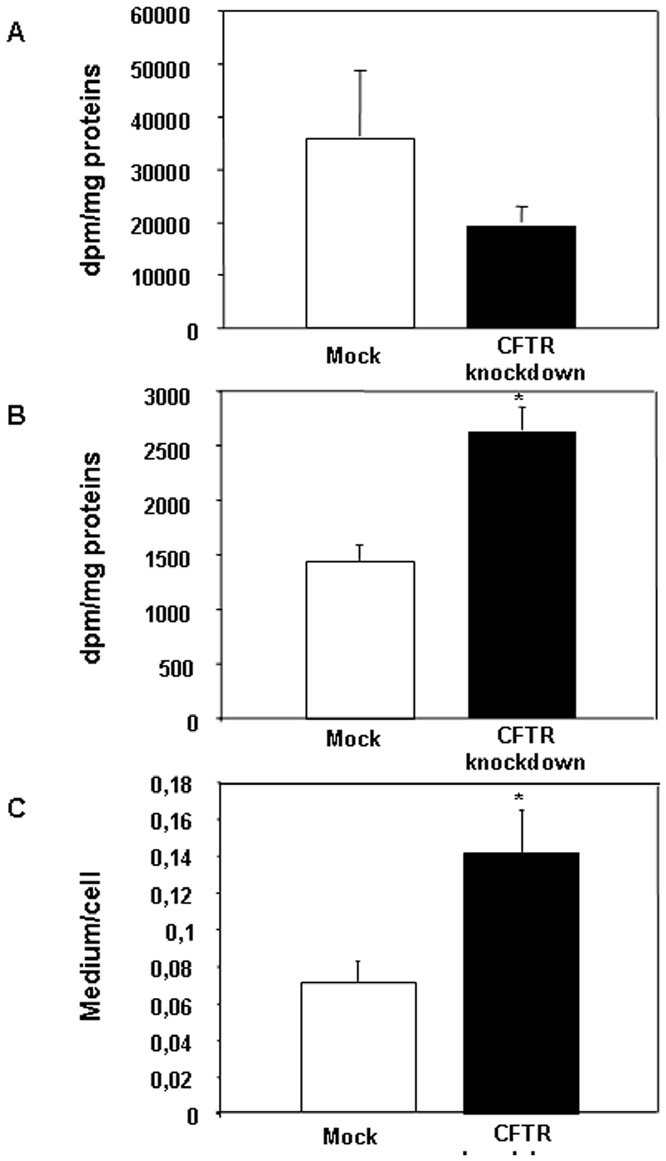
Impact of CFTR knockdown on newly synthesized (A), secreted (B) free fatty acid (FFA) and on the medium to cell ratio (C). After a 24 h-incubation with [^14^C]-acetate, cells and media were collected, lipids were extracted in chloroform/methanol and separated by TLC. The FFA band was scraped off the plate and counted for the amount of radioactivity incorporated. Data represented means ± SEM of n = 12 wells from two independent experiments. *p<0.05 vs mock cells.

We next examine the possibility that *de novo* lipogenesis could be stimulated in a state of CFTR depletion. To verify this hypothesis, we first incubated cells with [^14^C]-acetate, a major substrate for *de novo* lipogenesis and we assessed the amount of radioactivity incorporated into various lipid fractions in cell homogenates and in media. The amount of newly synthesized cellular lipids remained similar between control and genetically modified cells (Figure 7A). The major difference was, however, noted in the total amount of secreted lipids, which exhibited a 1.8-fold increase in CFTR knockdown cells (Figure 7B). This increase was largely attributed to the rise in the levels of PL, TG and CE. Consistent with the findings obtained with [^14^C]-oleic acid experiments, CFTR knockdown cells displayed higher levels of secreted FA than mock-control cells following [^14^C]-acetate incubation whereas cellular level of FFA exhibited a downward tendency at the expense of increased secretion documented by the augmented output of PL, TG and CE (Figure 8). These findings were further substantiated by the calculation of medium-to-cell ratios for each lipid fractions (Figure 7C and 8C). Significant higher ratios were found for all fractions following CFTR depletion, which indicated a marked lipid secretion to the medium.

**Figure 9:**
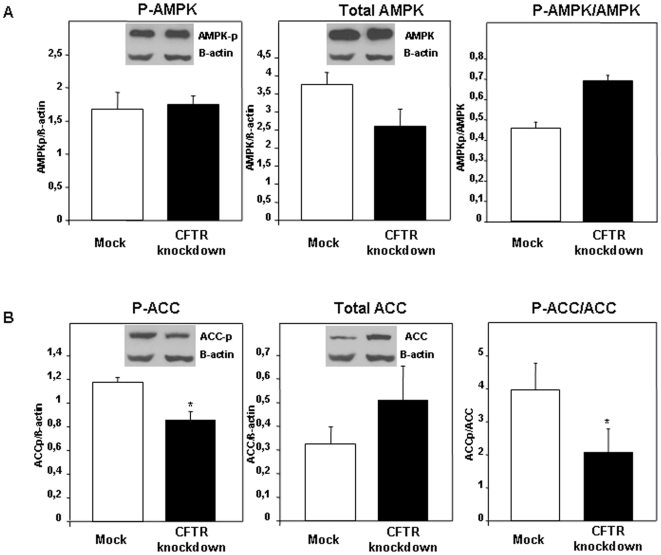
Impact of CFTR knockdown on the protein expression of (A) AMP-activated protein kinase (AMPK) and (B) acetylCoA carboxylase (ACC) in Caco-2/15 cells. Phosphorylated AMPK (p-AMPK) and ACC (p-ACC) and total AMPK and ACC were analyzed by Western Blot and the ratios of phosphorylated form to total protein level were calculated. Results are expressed as means ± SEM of n = 3 independent experiments. *p<0.05 vs mock cells.

**Figure 10:**
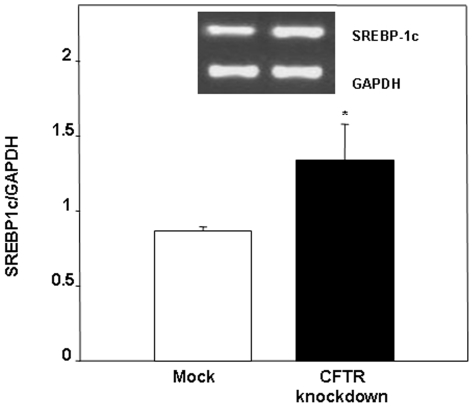
Expression of sterol regulatory element binding protein-1c (SREBP-1c) in response to CFTR knockdown in Caco-2/15 cells. PCR analysis was performed on cells differentiated for a period of 15 days. Y-axis represents SREBP-1c expression normalized to GAPDH expression. Results are expressed as means ± SEM of n = 5 independent experiments. *p<0.05 vs mock cells.

The mechanisms for the modifications of *de novo* lipogenesis were further investigated by examining key regulatory protein players involved in this metabolic pathway. We focused on AMPK that currently comes into a spotlight as an important regulator for lipid metabolism by modulating phosphorylation and activity of the key ACC enzyme. To this end, we performed Western blot analysis to estimate AMPK and ACC mass and phosphorylation (p-AMPK and p-ACC) in control and CFTR knockdown cells. CFTR-depleted cells displayed lower AMPK levels than controls without significant changes in the p-AMPK form, thereby resulting in a higher, although non-significant, p-AMPK/AMPK ratio (Figure 9A). On the other hand, we observed a significant decrease in the amount of p-ACC upon CFTR genetic manipulation, which was coupled to an increased trend of total ACC, leading to a marked decrease in the calculated ratio of p-ACC/ACC (Figure 9B). These data are suggestive that CFTR knockdown leads to a stimulation of the active form of ACC at the cellular level, thereby providing a molecular explanation for the enhancement in *de novo* lipogenesis. Since the induction of ACC gene transcription is under the regulatory action of SREBP-1c that binds the sterol response elements located in the ACC promoter, we determined its gene expression. In line with the enhanced lipogenesis and the decreased phosphorylation state of ACC, CFTR knockdown resulted in a significant increase in SREBP-1c gene expression (Figure 10).

### Gene expression of transcription factors

**Figure 11:**
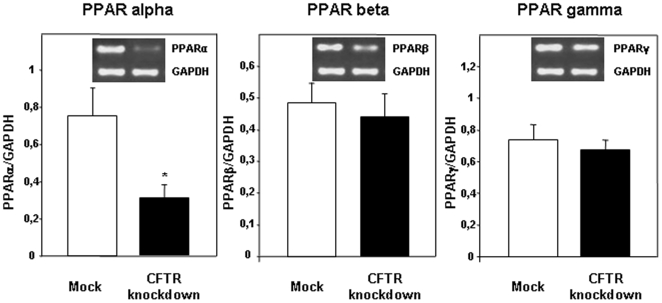
Effect of CFTR knockdown on peroxisome proliferators-activated receptors PPAR(α,β, γ) gene expression. Transcript levels were determined by RT-PCR using RNA extracted from Caco-2/15 cells differentiated for a period of 15 days. Results are expressed as means ± SEM of n = 5 independent experiments. *p<0.05 vs mock cells.

**Figure 12:**
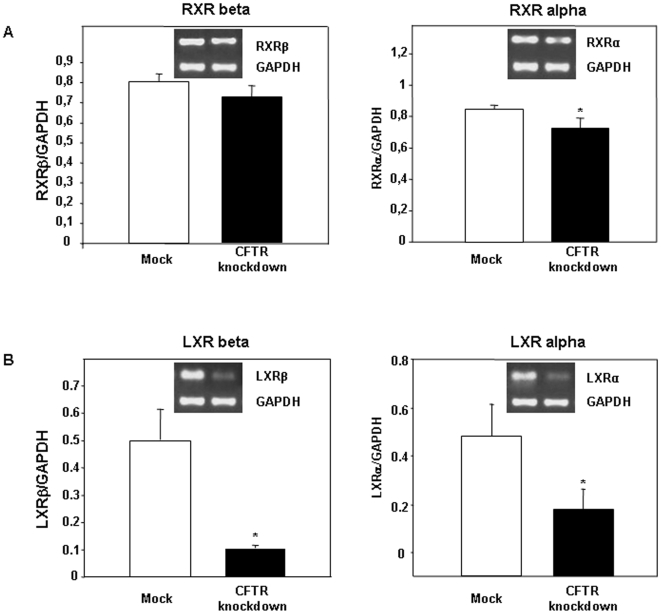
Effect of CFTR knockdown on gene expression of the nuclear receptors (A) retinoid X receptor (RXR) and (B) liver X receptor (LXR). Transcript levels were assessed by RT-PCR using RNA extracted from Caco-2/15 cells differentiated for a period of 15 days. Results are expressed as means ± SEM of n = 5 independent experiments. *p<0.05 vs mock cells.

In an attempt to elucidate potential mechanisms linking CFTR to FA metabolism, we determined the status of several transcription factors that govern the expression of a variety of genes associated with lipid and cholesterol metabolism. Data regarding the gene expression of members of the PPAR family are presented in Figure 11. CFTR disruption did not influence the expression of PPAR-β and PPAR-γ whereas a 2-fold reduction was observed in PPAR-α mRNA levels. Consistent with these data, a significant transcript decline characterized RXR-α (acting as a partner of PPAR-α), while the gene expression of RXR-β remained unaltered (Figure 12A). The most striking observation, in response to CFTR knockdown, was the downregulation of LXR-α and LXR-β mRNA (Figure 12B).

## Discussion

The mechanisms involved in the regulation of FA metabolism by CFTR are largely unknown despite recurrent evidences of abnormal FA composition and lipid profile in CF patients and heterozygous carriers of CFTR mutations [5], [7]. If numerous studies have examined the FA status in various cell types derived from CF patients, very few investigations have focused on the functional role of CFTR [3], [6]. Recent observations made by our group have suggested the implication of the chloride channel CFTR in the modulation of intestinal lipid handling and transport [23]. The present work considerably confirms and extends this concept by highlighting the impact of partial CFTR deletion on the composition, uptake and biogenesis of FAs in intestinal Caco-2/15 cells. In particular, we could document that disruption of CFTR expression led to a higher cellular FA content characterized by an elevated proportion of saturated and n-7 FA. We showed that the mechanism for the elevation of FA concentrations was mainly attributable to increased *de novo* lipogenesis. We have provided direct evidence for a role of ACC-mediated FA synthesis in line with SREBP-1c upregulation. Our results also indicate the relationship of CFTR depletion with enhanced FA uptake and transport via different lipoprotein classes. Finally, specific transcription factors involved in the regulation of lipid metabolism are strongly suppressed in the presence of reduced CFTR expression.

The present findings are in line with many other studies showing that plasma fractions of CF patients display elevated concentrations of 14:0, 16:0 and 16:1n-7 FA whereas 18:0 FA levels remain unchanged or slightly decreased [7], [34]. However, plasma FA largely represented a transport pool rather than a functional pool, which therefore underscores the importance of studying FA composition of CF-affected tissues and cells. Various studies performed on human biopsies [5], postmortem tissues [35], animal [4], [36] and cellular models [2], [3], [6] have reported FA abnormalities especially in the PUFAs. Mechanisms underlying these perturbations are still unclear but may be linked to dietary fat malabsorption and/or altered FA metabolism such as increased β-oxidation or lipid peroxidation as a consequence of oxidative stress, altered desaturase activity, enhanced production of eicosanoids and defective CFTR. However, data on intestinal FA distribution remained scarce and have been mostly reported in CF mice model with diverging conclusions attributable to confounding variables such as age, types of mice models and diets [4], [37], [38]. Using the intestinal Caco-2/15 cell model, we could show that, reduced CFTR expression distinctively affects the different classes of FA. While it exerts no particular influence on PUFAs, it increases the levels of n-7 FA whereas the effect on saturated FA family displays two opposed subclasses: saturated FA with 17 carbons and less exhibit higher concentrations in CFTR knockdown compared to control cells whereas longer saturated FA display reduced levels. Although, this dichotomy had recurrently been demonstrated in the plasma of CF patients [7], [33], the biological relevance and the mechanisms underlying these changes have been rarely explored and discussed despite the fact that they may be highly suggestive of abnormalities in tissue FA metabolism. Calculation of the desaturase ratios corroborates the assumption that FA metabolism is indeed altered in CFTR-depleted cells. Product-to-substrate ratio is good surrogate marker for desaturase activity in blood and tissue [33], [39]. Delta-7 and -9 desaturases catalyze the conversion of 16:0 into 16:1n-7 and 18:0 into 18:1n-9, respectively, the latter being a major constituent of TG. It has been reported that induction of delta-9 desaturase activity is observed in situations characterized by an increased demand for unsaturated FA [40]. We previously showed that CFTR-depleted cells exhibited enhanced TG esterification and lipoprotein assembly, both requiring unsaturated FA species [23]. Strong induction of delta-7 and -9 desaturase activities in knockdown cells may, therefore, be secondary to enhanced cellular requirement for unsaturated FA species. Although, there was no statistical differences between mock and CFTR knockdown cells in term of n-6 FA concentrations, CFTR-depleted cells displayed a lower delta-6 desaturase index suggesting impaired conversion of 18∶2, n-6 to more desaturated products. In contrast, treatment with the CFTR inhibitor, CFTR inh-172, led to an increase in all desaturase indexes (data not shown), which is concordant with a previous study performed in CF patients [33]. These observations suggest that the reduction of cellular content of CFTR protein exerts an impact on n-6 FA desaturation quite distinct from that observed when chloride conductance is blocked. Additional studies are necessary to delineate the mechanisms and increase our understanding on the role of CFTR protein content and activity in lipid transport and metabolism.

Deeper explorations of FA uptake and transport, using the monounsaturated [^14^C]-oleic acid, demonstrates that CFTR-depleted intestinal cells exhibit enhanced uptake of oleic acid, which translates in its increased secretion in the unesterified form. These findings are further substantiated by the enhanced L-FABP expression and a trend toward increased expression of the FA membrane transporter, FAT/CD36. L-FABP involvement in FA uptake has been reported in numerous investigations. Mice treated with clofibrate, a known activator of L-FABP, displayed enhanced liver FA uptake, intracellular diffusion and efflux [41]. Moreover, L-FABP disruption was associated with the stimulation of FA uptake both in fibroblasts and in l-fabp null mice [42], [43].

Under normal physiological conditions, the metabolic fate of FFA is to participate in sequential esterification reactions leading to the formation of TG, PL and CE that are subsequently packaged into lipoproteins and exported from the enterocyte, thereby explaining why lipoproteins carry very little amount of non-esterified FAs. Non-esterified FAs escaping the esterification pathway predominantly enter blood circulation bound to albumin or served to fuel mitochondrial β-oxidation. The high medium-to-cell ratios observed in CFTR knockdown cells demonstrated that these cells are particularly efficient at exporting lipids thereby strengthening the fact that lipid accretion acts as a trigger for stimulation of lipid transport. However, apart from increasing its buffer capacity through upregulation of L-FABP expression, Caco-2/15 cells appear to challenge the FA overload by efficiently channeling FAs toward the lipoprotein secretory pathways, i.e. the endoplasmic reticulum. It has been shown in several cellular models that the exposition to high concentrations of saturated and unsaturated FA promotes TG synthesis and prevents lipotoxicity by sequestering FFA that are otherwise cytotoxic [44]. In the present case, increased lipoprotein content of FFA represents a novel and non-classical pathway that may fulfill the same role of protecting the cell against FA overload. Since CFTR knockdown cells exported more lipids than mock cells but still exhibited a higher cellular FA content, we speculate that CFTR depletion leads to an inappropriate homeostatic response to FA accumulation where the increasing amount of cellular FA is preferably incorporated and secreted into lipoproteins. Reduction in CFTR expression appears to cause a shift in the balance between energy-yielding and energy-consuming reactions in favor of the latter. To support this claim, we found that, when CFTR-depleted cells were incubated with [^14^C]-acetate, the major lipogenic substrate, they displayed an enhanced output of newly synthesized FFA, TG, PL and CE compared to mock-infected cells. These findings were further confirmed by the assessment of p-ACC protein and SREBP-1c mRNA levels. ACC is the key enzyme catalyzing the conversion of acetyl CoA into malonyl CoA which is subsequently converted to palmitate under the control of the FA synthase whereas SREBP-1c is a transcription factor involved in the control of the expression of lipogenic genes. In CFTR-depleted cells, the mass of p-ACC, the inactive form of the protein, was significantly reduced resulting in a decreased p-ACC to total ACC ratio indicative of increased activity. Consistent with these data, SREBP-1c gene expression was also increased. Likewise, a recent study has reported an increased SREBP-1c expression and activity in CF dendritic cells [45]. Collectively, these data support a role for CFTR in the control of intestinal lipogenesis. However, additional studies are clearly needed to clarify the impact of CFTR depletion on the FA oxidation status especially in view of a recent report suggesting an impairment of β-oxidation in CF patients [46].

Why would intestinal cells, not recognized for lipid storage, exhibit enhanced lipogenesis in a state of CFTR depletion? Moreover, why would CFTR knockdown cells demonstrate increased lipogenesis and FA uptake, two processes leading to cellular FA accretion and stimulation of FA incorporation and secretion into lipoproteins? Although counterintuitive, this situation is not unique to our model and resembles in many aspects to what is observed in enterocytes isolated from fructose-fed hamster, an animal model of insulin resistance. In this model, overproduction of intestinal apolipoprotein B48-containing lipoprotein was closely associated with stimulation of intestinal *de novo* lipogenesis [47].

Genetic and pharmacological manipulation of CFTR led to comparable changes in cellular FA profile, which strongly indicates that CFTR plays a role in the regulation of intestinal FA metabolism. The changes observed in the presence of the inhibitor were, however, less pronounced than those induced by CFTR deletion, thereby suggesting that blocking chloride conductance is one of the prerequisites for perturbations of intestinal FA metabolism. Even if blocking chloride conductance was previously shown to disturb the FA profile of airway epithelial cells [22], reduction in the cellular content of CFTR appears to exert additional influences. In fact, lack of CFTR at the plasma and subcellular membranes may affect biophysical properties, thereby culminating in alterations of various functions, including lipid synthesis and transport. Furthermore, CFTR depletion may hamper the potential interactions with other proteins, including transporters, receptors and signalling proteins.

Using several experimental approaches, our data strengthen the notion that CFTR is involved in the regulation of intestinal FA metabolism through modulation of the lipogenic pathway. Previous evidence has suggested that CFTR interacted with AMPK, which is involved in the regulation of cellular energy homeostasis [48], [49]. AMPK activity is stimulated when the intracellular energy status is low (i.e increased in AMP/ATP ratio). The action of AMPK is aimed at increasing the production of ATP by enhancing FA oxidation and inhibiting FA biosynthesis through p-ACC. ACC deactivation leads to carnitine palmitoyltransferase inhibition, thereby facilitating mitochondrial FA uptake and catabolism. Since AMPK colocalizes with CFTR and given that both entities appear to exert an influence on each other [48], [49], it is tempting to postulate that CFTR depletion would enhance lipogenesis through modulation of AMPK activity. This hypothesis turns out to be false. Levels of p-AMPK were not significantly altered in the CFTR depletion condition whereas the mass of total AMPK was significantly reduced resulting in an upward, albeit moderate, shift of the p-AMPK to total AMPK ratio, an index of AMPK activity. These results were unexpected giving that numerous studies have reported that p-ACC remained under the control of AMPK. Dissociation between activation of AMPK and p-ACC has been documented in ischemic kidneys [50] as well as in muscle following prolonged exercise [51]. More importantly, defective FA oxidation has been suggested in CF [46], which is in agreement with the idea of an uncoupling between AMPK activity and the control of FA anabolic and catabolic pathways under a state of CFTR depletion.

Our data showed a reduction in the expression of several transcription factors involved in the control of cholesterol and FA metabolism in CFTR-depleted cells. PPARα expression, whose activation is normally induced by PUFA, was markedly suppressed in CFTR knockdown cells despite unchanged cellular levels of n-3 and n-6 FAs. The LXR family of transcription factors regulates a wide array of genes, including SREBP-1c and ACC. Surprisingly, CFTR depletion leads to the suppression of the gene expression of both LXR members, therefore suggesting that regulation of SREBP-1c and ACC occurs in a LXR-independent manner. This situation is very similar to the effects of a high-glucose diet, which induces, even in the absence of LXR, liver expression of several lipogenic genes, including ACC [52]. The mechanisms underlying the suppression of PPARα, LXRs and RXRα and their relation CFTR depletion are still unknown and need to be further investigated.

High concentrations of TG and fasting hypertriglyceridemia are frequently encountered in CF patients [53]. The reasons underlying these conditions are not well understood but have been related to chronic low-grade inflammation and imbalance in dietary macronutrients intake in favor of carbohydrates [53]. Increased intestinal synthesis of CM and of their major protein component, apolipoprotein B48, have been identified as important contributors to the dyslipidemia associated with insulin-resistant and diabetic states [47], [54], [55]. As demonstrated previously, CFTR depletion in Caco-2/15 cells has led to the enhancement of apolipoprotein B biosynthesis and the assembly of CM. Furthermore, the present findings have revealed that CFTR reduced expression is associated with increased *de novo* lipogenesis. Therefore, we can speculate that the higher-than-normal TG levels documented in CF patients might be the result of impaired intestinal lipid metabolism consequent to CFTR depletion.

CF patients are instructed to consume a high calorie, high fat diet to lessen the detrimental effects of lipid malabsorption that persists despite tailored pancreatic enzyme supplementation. Etiology of malnutrition in CF patients includes factors such as maldigestion, malabsorption, abnormal mucous secretions, poor appetite, abdominal pain, gastro-esophageal reflux and anorexia secondary to chronic infections [56]. Our findings have, at least, contributed to exclude CFTR as a causative factor for defective mucosal uptake and intracellular transport of long-chain FA as well as impaired intra-enterocyte handling of lipids. Of interest, our previous studies reported the significant contribution of the permanent essential FA deficiency to CF malabsorption by interfering with intra-enterocyte lipid transport [15], [57].

We have examined how genetic manipulation of CFTR level affects Caco-2/15 cell integrity (data not shown). No alterations were noted in cell viability between control Caco-2/15 cells, mock cells and Caco-2/15 cells deficient in CFTR. Similarly, estimation of the expression of the two characteristic brush border membrane protein markers, villin and sucrase, did not reveal significant changes, which suggests that genetically modified Caco-2/15 cells are viable and fully differentiated. Moreover, we assessed transepithelial electrical resistance, a reliable indicator of the tightness of the junctions between epithelial cells, which represents an accurate determination of paracellular permeability [58], [59]. Once again, no differences were observed between mock and knockdown cells, which indicate that CFTR lessening has not influenced paracellular permeability of oleic acid.

**Figure 13:**
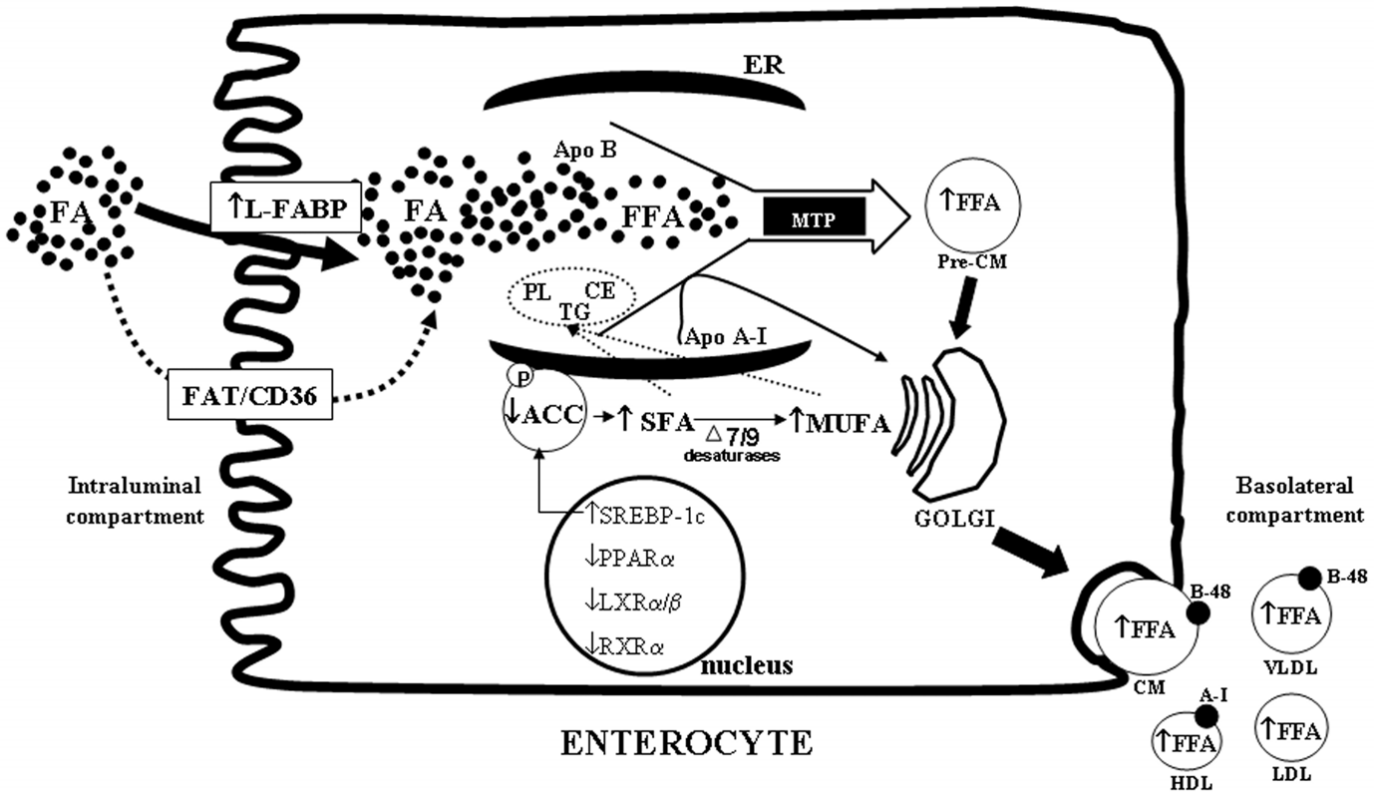
Schematic representation of the different cellular changes induced by CFTR knockdown in Caco-2/15 cells. CFTR depletion led to an increase in FA uptake mostly by L-FABP (and to a lesser extent by CD36) and channeled to the lipoprotein assembly pathway, resulting in increased output of FFA-enriched lipoproteins. Furthermore, activation of the lipogenic pathway through stimulation of SREBP-1c expression and ACC (dephosphorylation and activation) caused cellular FA accretion, especially SFA which were converted to MUFA due to increased desaturase activity. These newly synthesized FA were incorporated into PL, TG and CE and efficiently secreted by the cell. ACC: acetyl-CoA carboxylase; Apo: apolipoprotein; CE: cholesteryl ester; CM: chylomicron; FFA: free fatty acid; HDL: high-density lipoprotein; L-FABP: liver fatty-acid binding protein; LDL: low-density lipoprotein; LXR: Liver X receptor; MTP: microsomal transfer protein; MUFA: monounsaturated fatty acid; PPAR: Peroxisome proliferators-activated receptor; PL: phospholipids; RXR: Retinoid X receptor; SFA: saturated fatty acid; SREBP-1c: Sterol regulatory element-binding protein-1c; TG: triglycerides; VLDL: very low-density lipoprotein.

In conclusion, our data bring novel and exciting insights on the effects of CFTR depletion, at a level seen in heterozygous subjects and even individuals carrying other CFTR mutations, on intestinal FA homeostasis. As depicted in Figure 13, CFTR-depleted cells exhibited significant alterations in FA metabolism as exemplified by the accumulation of cellular FA, especially saturated and monounsaturated classes, enhancement of FA uptake and transport and stimulation of the lipogenic pathway through LXR/RXR-independent mechanisms. Modifications in the FA profile were reproduced to a lesser extent when CFTR was pharmacologically blocked with the selective inhibitor, CFTR-inh 172 suggesting a direct involvement of CFTR *per se*. Changes in intestinal FA metabolism related to defective CFTR may have deleterious health consequences for CF patients and may explain certain aberrations in plasma saturated and n-7 FA and pathophysiological manifestations such as hypertriglyceridemia.
