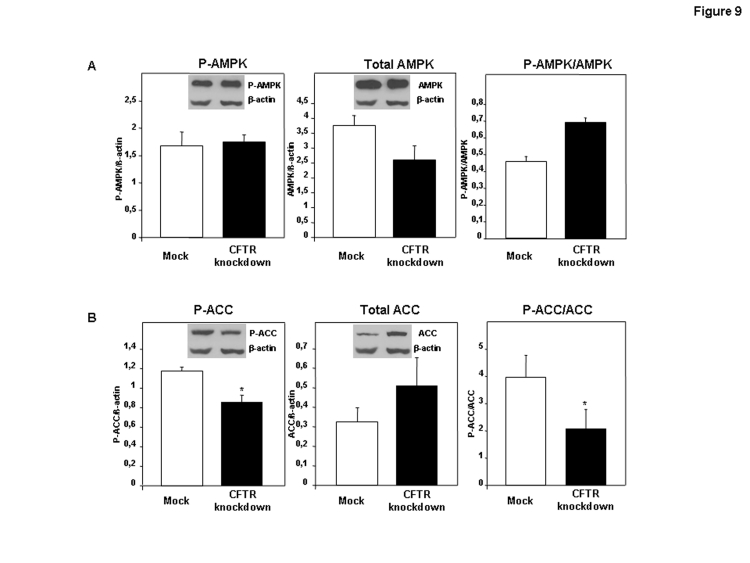# Correction: CFTR Depletion Results in Changes in Fatty Acid Composition and Promotes Lipogenesis in Intestinal Caco 2/15 Cells

**DOI:** 10.1371/annotation/f9b8a9d2-4be3-4981-92f4-a3b4cb0b0bf5

**Published:** 2010-05-28

**Authors:** Geneviève Mailhot, Rémi Rabasa-Lhoret, Alain Moreau, Yves Berthiaume, Emile Levy

In Figure 9, the X-axes of 4 graphs are incorrect. Please view the correct figure here: 

**Figure:**